# Delayed Post-Operative C5 Palsy After Reduction of Unilateral Cervical Facet Dislocation: A Case Report

**DOI:** 10.7759/cureus.80429

**Published:** 2025-03-11

**Authors:** Dillon C Benson, Christopher Johnson, Michael Lee, Mostafa El Dafrawy

**Affiliations:** 1 Department of Orthopaedic Surgery and Rehabilitation Medicine, University of Chicago Medical Center, Chicago, USA

**Keywords:** c5 palsy, cervical spine injury, post-operative c5 palsy, “post-operative complications”, traumatic cervical spine injury

## Abstract

A 60-year-old female sustained a left unilateral C3-4 facet dislocation and underwent urgent open reduction and posterior instrumentation. The patient subsequently developed delayed onset right upper extremity weakness affecting the C5 myotome, prompting a diagnosis of post-operative right C5 palsy (C5P). With conservative management, the patient had a complete neurologic recovery in eight months. Surgeons must be aware of the development of post-operative C5P whenever operating on the cervical spine, regardless of the operative level or procedure performed. This is the first documented case of C5P after the reduction of a facet dislocation, which occurred after a procedure not involving the C4-5 level.

## Introduction

C5 palsy (C5P) is a well-documented complication following cervical spine surgery; however, its cause remains controversial. Post-operative C5P was first described in 1965, with numerous subsequent studies citing an incidence of C5P ranging from 0% to 30%, with a recent systematic review citing a pooled prevalence of 6.0% [1-4]. Various pathophysiologic hypotheses have been suggested, including direct nerve root injury, nerve root ischemia, reperfusion injury following decompression, post-decompression nerve root tethering, spinal cord rotation, and traction injury from dorsal spinal cord migration [4].

The predominance of literature covering the topic of post-operative C5P describes its occurrence following cervical decompression [3-7]. This case illustrates its development following the reduction of a unilateral cervical facet dislocation with no decompression performed. This case report offers further insight into the various mechanisms in which the clinical presentation of C5P may result.

The patient was informed that data concerning the case would be submitted for publication, and she provided consent.

## Case presentation

**Figure 1:**
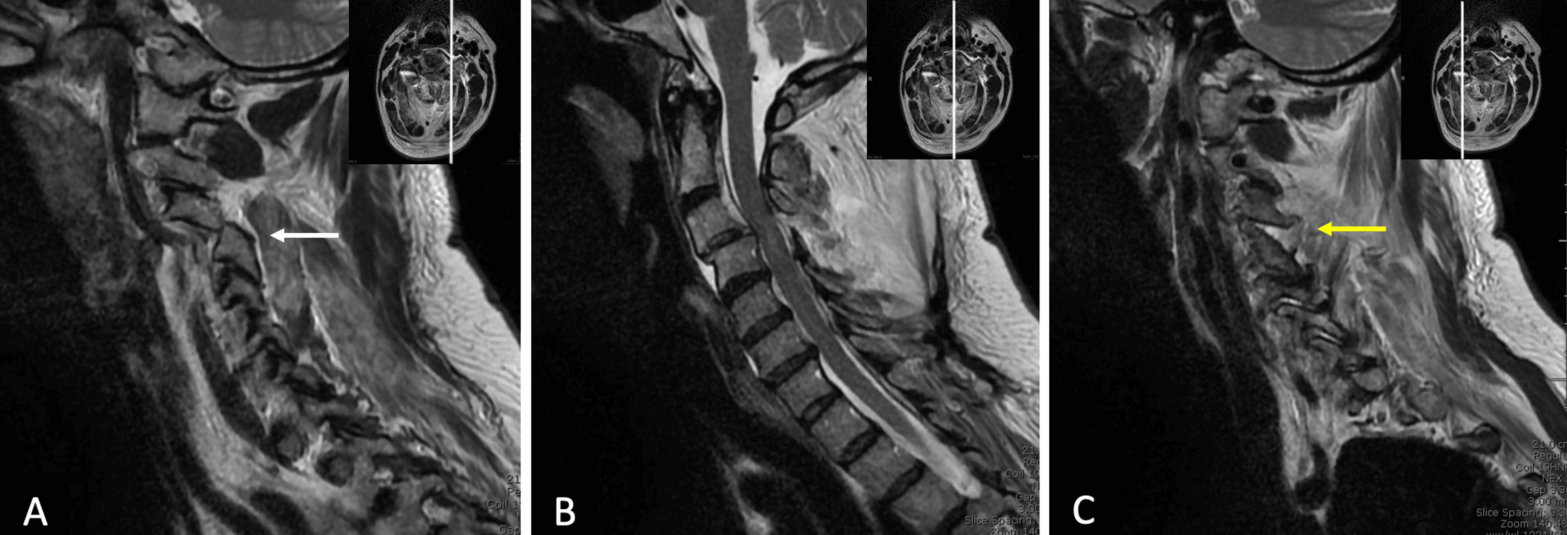
(A,B,C) Pre-operative magnetic resonance imaging (MRI) of the cervical spine demonstrating a left unilateral C3-4 facet dislocation (white arrow). The right parasaggital image (C) demonstrates the distracted right C3-4 facet (yellow arrow).

A 60-year-old female presented as an outside hospital transfer after being thrown from a horse. When examined upon presentation, she was found to have cervical spine tenderness, decreased sensation to light touch and pinprick to her right thumb, bilateral shoulder hyperesthesias, and decreased motor strength in bilateral upper extremity deltoids (4+/5) and biceps (4+/5). The remainder of the neurologic exam was without deficit. A computed tomography (CT) and subsequent magnetic resonance imaging (MRI) of her cervical spine demonstrated a left unilateral C3-4 facet dislocation (Figure 1). 

**Figure 2:**
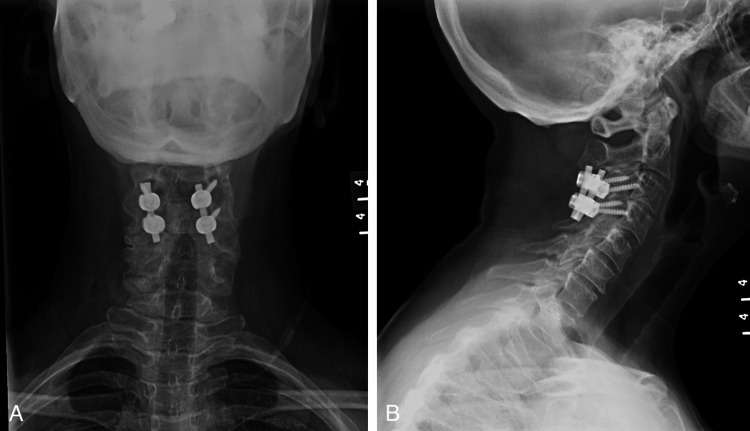
(A, B) Post-operative radiographs obtained at the one-year post-operative visit demonstrating reduced C3-4 facets with C3-4 posterior instrumentation.

She was urgently taken to the operating room for an open reduction and posterior instrumented fusion (Figure 2). A high-speed burr was used to remove the superior portion of the C4 left superior facet, allowing for subsequent reduction. The patient’s immediate post-operative course was uneventful. Her neurologic examination remained consistent with her pre-operative examination. The patient was discharged on post-operative day three. 

On post-operative day seven, the patient reported progressive right arm weakness without pain and without sensory changes. Upon examination, she was found to have 2/5 deltoid and 3/5 bicep motor strength, while her left upper extremity motor strength had improved to 5/5 throughout. She denied any pain or any sensory changes from her initial clinical presentation. Based on this exam and upright X-ray imaging unchanged from immediate post-operative imaging, she was diagnosed with post-operative C5 palsy.

At her two-week post-operative appointment, an examination of her right upper extremity demonstrated 2/5 deltoid and 4-/5 bicep motor strength. The sensory examination had subjective improvement but was objectively unchanged from the pre-operative exam. She continued to report no pain. She remained engaged in physical therapy throughout her post-operative course.

At her six-week post-operative appointment, an examination of her right upper extremity demonstrated 3/5 deltoid and 4/5 bicep motor strength. However, her sensation changes at the time of surgery had completely resolved.

By eight months post-injury, she had a complete neurologic recovery with full upper extremity motor strength and no altered sensation. At one year post-injury, she continued to have no neurologic deficits. 

## Discussion

Post-operative C5 palsy is characterized by the onset of C5 motor weakness after cervical spine surgery [6]. Significant variations in presentation exist, including whether it is sensory sparing or not, painful or painless, severity of weakness, timing of symptom onset, and recovery. Presentations have been described to occur immediately after surgery as well as delayed in onset up to two weeks following the procedure [7]. While well studied, there has not been a consensus definition or classification reached. The heterogeneous presentation and lack of clear diagnostic criteria have led to an inability to garner meaningful clinical conclusions from much of the research [8].

In a systematic review of risk factors for post-operative C5 palsy, Jack et al. found that decreased foraminal diameter and pre-operative cord rotation were associated with post-operative C5P [4]. Risk factors identified in other studies include ossification of the posterior longitudinal ligament, posterior surgical approach, C5 foraminotomy, and corpectomy [5,6,9].

The pathophysiologic mechanisms for post-operative C5 palsy are poorly understood. They include direct nerve root injury, nerve root ischemia, reperfusion injury following decompression, post-decompression nerve root tethering, spinal cord rotation, and traction injury from dorsal spinal cord migration [4,7]. They have occurred after posterior and anterior approaches. Multiple authors have recommended a distinction based on the timing of symptom onset, as direct nerve injury, nerve root ischemia, and reperfusion injury have an immediate or early post-operative onset, while the other mechanisms mentioned have the more classically associated delayed onset [8,10]. Whether due to tethering, cord rotation, or traction injury, basic science studies have demonstrated how small increases in nerve strain can lead to clinically significant conduction abnormalities. Wall et al. showed that at only 6% nerve strain, there is a 70% decrease in conduction amplitude, which reaches a complete conduction block with minimal recovery at 12% nerve strain [4,11].

Our patient’s facet dislocation and stabilization occurred at the C3-4 level, not the C4-5 level. To our knowledge, there are no prior reports of delayed post-operative C5 palsy occurring after the reduction and stabilization of a facet dislocation. Furthermore, there are few examples of post-operative C5 palsy occurring after a surgery not performed at the C4-5 level [12]. This case further highlights the incomplete understanding of the etiology of post-operative C5P. A possible explanation for the C5 palsy occurring after a surgery performed at C3-4 would be the presence of a pre-fixed brachial plexus. Additionally, C5P is not an isolated complication of cervical decompression, illustrated by the fact that no decompression was performed in this case and may occur following any cervical spine surgery, including reduction of a facet dislocation.

Despite the various proposed etiologies and presentations of post-operative C5P, it remains reassuring that most patients experience neurologic improvement, and 41% of patients achieve a full recovery [9,13]. It is imperative to discuss the timeline of recovery when counseling patients. As experienced by our patients, recovery often takes up to six months; however, it can take up to 12 months for patients to reach their new functional baseline [9,13,14]. Pennington et al. demonstrated that as the severity of motor deficit increased, so too did the time to neurologic recovery [13].

## Conclusions

This case illustrates that surgeons must be aware of the development of post-operative C5P whenever operating on the cervical spine, regardless of the operative levels or procedure performed. The unique aspects of this presentation are its occurrence following the treatment of a unilateral facet dislocation and at a non-C4-5 level. No cases in the current literature describe a post-operative C5P following reduction and instrumentation of a unilateral cervical facet dislocation. This case report adds to the current body of evidence that there is not a single mechanism of injury responsible for C5P, but that C5P is a clinical manifestation that may occur as a result of multiple possible insults to the C5 nerve root.
